# Factors associated with fibrotic-like pattern on thorax CT after COVID-19 pneumonia

**DOI:** 10.5578/tt.2025011031

**Published:** 2025-03-24

**Authors:** Miraç ÖZ KAHYA, Aslıhan GÜRÜN KAYA, Övgü VELİOĞLU YAKUT, Sema Nur DOĞRU, Serhat EROL, Fatma ARSLAN, İrem AKDEMİR, Güle ÇINAR, Çağlar UZUN, Neriman Defne ALTINTAŞ, Aydın ÇİLEDAĞ, Kemal Osman MEMİKOĞLU, Akın KAYA, Öznur YILDIZ, Oya KAYACAN, Özlem ÖZDEMİR KUMBASAR, Sevgi SARYAL

**Affiliations:** 1 Department of Chest Diseases, Ankara University Faculty of Medicine, Ankara, Türkiye; 2 Department of Infectious Diseases and Clinical Microbiology, Ankara University Faculty of Medicine, Ankara, Türkiye; 3 Department of Radiology, Ankara University Faculty of Medicine, Ankara, Türkiye; 4 Division of Intensive Care, Department of Internal Medicine, Ankara University Faculty of Medicine, Ankara, Türkiye

## Abstract

**ABSTRACT**

**
Factors associated with fibrotic-like pattern on thorax CT after COVID-19 pneumonia
**

**Introduction:** *This study aimed to investigate whether coronavirus disease-2019 (COVID-19) leads to impaired pulmonary function, fibrotic-like abnormalities or psychological symptoms six months after discharge.
*

**Materials and Methods:** *This study involves 162 laboratory-confirmed patients with COVID-19 who were diagnosed in Ankara University Faculty of Medicine, Department of Chest Diseases from February 1, 2021 to July 1, 2023. All patients were diagnosed with COVID-19 pneumonia by thorax computed tomography (CT). Patients who applied to the outpatient clinic six months after COVID-19 treatment were included in the study. A total of 133 patients underwent thorax CT scan, pulmonary function tests, six minutes walking test simultaneously. Radiographic patterns were categorized into two groups (normal/non-fibrotic and fibrotic-like). Group A had 66 patients who either had no fibrotic or non-fibrotic changes, and 67 patients who had fibrotic-like changes were categorized as group B.
*

**Results:** *Mean age of the study subjects was 55.95 ± 12.42 years, and 75 (56.4%) patients were male. Overall, median diffusing capacity of the lungs for carbon monoxide (DLCO) % predicted measured as 73.5% [IQR 61-88]. DLCO and six-minute walking distance were significantly lower in the fibroticlike pattern group (p< 0.001, p= 0.014, respectively). Reduced DLCO in patients with fibrotic-like pattern after six months was common. Presence of ground-glass opacities, reticulations and traction bronchiectasis correlated strongly with reduced diffusing capacity (r= -0.190 p= 0.043, r -0.305 p= 0.001, r -0.404 p< 0.001, respectively). We demonstrated that smoking history and intensive care unit (ICU) admisson during COVID-19 pneumonia were independent risk factors for fibrotic-like radiographic abnormalities.
*

**Conclusion:** *Residual abnormalities resembling fibrosis were notably prevalent, particularly among severely ill patients, and impaired lung diffusion persisted in some individuals even six months post-discharge. Post-COVID-19 lung sequelae can persist and progress after hospital discharge, suggesting airways involvement and formation of new fibrotic-like lesions, mainly in patients who had been in the ICU and had smoking history.
*

**Key words:** *CT imaging; follow-up; SARS-CoV-2; COVID-19; fibrosis, fibroticlike
*

**ÖZ**

**
COVID-19 pnömonisi sonrası Toraks BT'de fibrozis benzeri patern ile ilişkili faktörler
**

**Giriş:** *Bu çalışmada, koronavirüs hastalığı-2019’un (COVID-19) taburcu olduktan altı ay sonra solunum fonksiyon bozukluğuna ve toraks bilgisayarlı tomografisinde (BT) fibrozis benzeri değişikliklere yol açıp açmadığını araştırdık.
*

**Materyal ve Metod:** *Bu çalışma, 1 Şubat 2021-1 Temmuz 2023 tarihleri arasında Ankara Üniversitesi Tıp Fakültesi Göğüs Hastalıkları Anabilim Dalında laboratuvar ile doğrulanmış COVID-19 tanısı konulan 162 hastayı kapsamaktadır. Tüm hastalara toraks BT ile COVID-19 pnömonisi tanısı konulmuştur. Koronavirüs hastalığı-2019 tedavisinden altı ay sonra polikliniğe başvuran hastalar çalışmaya dahil edilmiştir. Toplam 133 hastaya toraks BT, solunum fonksiyon testleri ve altı dakika yürüme testi uygulanmıştır. Radyolojik paternler normal/fibrotik olmayan ve fibrozis benzeri patern olarak iki gruba ayrılmış- tır. Grup A’da normal veya fibrotik olmayan değişiklik gösteren 66 hasta, grup B’de fibrozis benzeri değişiklikler gösteren 67 hasta yer almıştır.
*

**Bulgular:** *Hastaların yaş ortalaması 55.95 ± 12.42 yıl olup, 75 (%56.4)’i erkekti. Genel olarak karbon monoksit difüzyon kapasitesi (DLCO) ortalama %73.5 [IQR 61-88] olarak ölçülmüştür. Grup B'de DLCO ve altı dakika yürü- me mesafesi anlamlı şekilde daha düşüktü (sırasıyla p< 0.001 ve p= 0.014). Altı ay sonra fibrozis benzeri paterne sahip hastalarda DLCO düşüşü daha sık gözlemlenmiştir. Buzlu cam opasiteleri, retikülasyonlar ve traksiyon bronşektazisi varlığı azalmış difüzyon kapasitesi ile ilişkili bulunmuştur (sırasıyla, r= -0.190 p= 0.043, r= -0.305 p= 0.001, r= -0.404 p< 0.001). Sigara öyküsü ve COVID-19 pnömonisi sırasında yoğun bakım ünitesine (YBÜ) kabul edilmenin fibrozis benzeri radyolojik bulgular için bağımsız risk faktörleri olduğu saptanmıştır.
*

**Sonuç:** *Özellikle ağır hastalarda fibrozise benzeyen sekel değişikliklerin yaygın olduğu ve bazı bireylerde altı ay sonrasında difüzyon kapasitesinde düşüşün devam ettiği gözlemlenmiştir. Koronavirüs hastalığı-2019 sonrası akciğer sekellerinin hastane taburculuğundan sonra da devam edebileceği ve ilerleyebileceği, özellikle YBÜ’de tedavi gören ve sigara öyküsü bulunan hastalarda yeni fibrozis benzeri lezyonların oluşabileceği akılda tutulmalıdır.
*

**Anahtar kelimeler:** *BT görüntüleme; takip; SARS-CoV-2; COVID-19; fibrozis, fibrozis benzeri görünüm
*

## INTRODUCTION

Coronavirus disease-2019 (COVID-19) pandemic represents a new disease, and long-term pulmonary outcomes of COVID-19 are unknown (1). As the identification of the first cases and the five-year course of the pandemic are now behind us, certain aspects of COVID-19 remain uncertain with long- term effects of the disease being one of the most prominent areas of ambiguity. Long-term effects are still unclear, making it difficult to determine which patients need standard follow-up to detect fibrosis in

time for new pandemic waves (2). Thorax computed tomography (CT) is important for diagnosis, treat- ment, and follow-up in patient with COVID-19 pneu- monia. Particularly, in the follow-up period, thorax CT is offered to evaluation the progress of disease and fibrotic changes in lungs (3). Early studies on survi- vors following hospitalization for COVID-19 indicate that nearly 50% exhibited reduced diffusing capacity, decreased total lung capacity (TLC), impaired exer- cise capacity, and/or abnormal thorax CT scans within one-month post-discharge (4). It remains uncertain whether the persistent pathological

findings on CT scans will evolve into symptomatic pulmonary fibrosis over time or whether different treatments for COVID-19 could impact the long-term resolution of parenchymal opacities. Follow-up stud- ies have identified impaired lung diffusing capacity for carbon monoxide (DLCO), TLC, and poor perfor- mance in six-minute walking test (6MWT) as the most prevalent lung function abnormalities. Our understanding of long-term consequences remain limited, making it challenging to identify which patients require routine follow-up to promptly detect fibrosis, especially in the event of future pandemic waves. In this study, we aimed to determine predic- tors of pulmonary fibrosis and early warning indica- tors that are linked to pulmonary fibrosis in treated patients with COVID-19 pneumonia. Our secondary aim was to evaluate if radiologic abnormalities and functional outcomes were associated with persistent impaired lung diffusion.

### MATERIALS and METHODS

**Study Design**

We conducted a single-center prospective cohort study of adults presented to outpatient clinic between February 1, 2021 and July 1, 2023 who had been treated for COVID-19 pneumonia at our hospital. The Ankara University Faculty of Medicine Ethical Committee’s protocol number is I1-15-21. Participants signed a written informed consent.

In this prospective study, patients diagnosed as COVID-19 pneumonia more than six months ago and presented to the outpatient clinic after being discharged following COVID-19 pneumonia were included.

Demographic data, smoking history, comorbidities, symptoms, laboratory and radiological findings at diagnosis and admission were examined. All data on diagnosis were obtained from hospital medical records retrospectively. Pulmonary function tests and thorax CT were performed simultaneously on the patients at the admission prospectively.

This study includes 162 patients diagnosed with COVID-19-associated pneumonia, all of whom had confirmed severe acute respiratory syndrome corona- virus-2 infection through a positive reverse-transcrip- tion polymerase chain reaction test performed on nasopharyngeal swab samples at the time of diagno- sis. These patients were followed up.

Inclusion criteria are shown in Table 1.

**Table:** 

**Table 1.** Inclusion criteria
Age ≥18 years Written informed consent Diagnosis of SARS-CoV-2 infection by positive PCR on the nasopharyngeal swab Presence of any radiological signs of COVID-19 pneumo- nia on thorax CT at admission Patients diagnosed and treated for COVID-19 pneumonia in our hospital Patients presented to the outpatient clinic six months after treatment for COVID-19 pneumonia Patients whose data can be accessed through hospital medical records
SARS-CoV-2: Severe acute respiratory syndrome coronavirus-2, PCR: Polymerase chain reaction, COVID-19: Coronavirus disease-2019, CT: Computed tomography.

At six month following COVID-19 diagnosis, 133 participants underwent a non-contrast thorax CT scan, pulmonary function tests (spirometry, lung dif- fusing capacity for carbon monoxide, lung volumes with nitrogen washout method), measurement of six- minute walking distance (6MWD). All participants were also evaluated for demographic and clinical features and laboratory parameters. The effect of pul- monary fibrosis on pulmonary function was analyzed simultaneously.

Study patients were divided into two groups accord- ing to thorax CT findings as normal/non-fibrotic pat- tern and fibrotic-like pattern.

### CT imaging

Non-contrast thin-section thorax CT scans were per- formed each patient using four-slices CT scanner (Toshiba Asteion 4, Toshiba Medical System, Japan) at diagnosis period and 16-slices multi dedector CT scanner (GE Light Speed, GE Medical System, Milwaukee WI) at sixth months. Images were acquired in supine position in the craniocaudal direction at end-inspiration.

CT images were evaluated for the presence of the following characteristics: Normal findings, intra- parenchymal bands, ground-glass opacities, consoli- dation, reticulations, traction bronchiectasis, and honeycombing. All thin-section CT images were independently analyzed by a radiologist to determine the presence of pulmonary fibrosis and fibrotic-like changes.

Fibrotic abnormalities on thorax CT imaging were defined as combination of findings including reticu- lation, traction bronchiectasis and honey-combing. Fibrotic-like patterns included those with subpleural reticulations, traction bronchiectasis or honeycomb- ing (5).

Radiographic patterns were categorized into two groups (normal/non-fibrotic and fibrotic-like). Group A had 66 patients who either had no fibrotic or had non-fibrotic changes; 67 patients who had fibrotic- like changes were categorized as group B. None of the patients had fibrotic pattern on thorax CT at sixth month.

### Pulmonary function tests

Pulmonary function testing (spirometry, lung volume and lung diffusing capacity for carbon monoxide) were performed in all participants by Vmax Encore 229 Pulmonary Function Germany-21.2 according to the guidelines of the American Thoracic Society (6,7). Pulmonary function parameters included forced vital capacity (FVC), forced expiratory volume in the first

second (FEV1), the FEV1/FVC ratio, DLCO, and TLC. The 6MWT was performed according to the current

American Thoracic Society guidelines (8).

### Statistical Analysis

For descriptive statistics, mean (standard deviation) or median (interquartile range) were used for quanti- tative variables with a normal or non-normal distribu- tion, respectively. Normality of the distribution was analyzed using the Kolmogorov-Smirnov test. The frequencies were used for qualitative variables. Continuous variables were presented as mean ± standard deviation, and categorical variables were presented as n (%). We examined associations between clinical and laboratory characteristics with no abnormalities/non-fibrotic pattern and fibrotic- like pattern. Pearson’s chi-square test and Fisher’s exact test were used to analyze categorical data as appropriate. Median scores of the two groups were compared with non-parametric Mann-Whitney U test. The student’s t-test for unpaired data was used to compare parametric variables.

We calculated correlations between continuous and ordinal data using Pearson point biserial correlation method. A 5% type-I error level was used to infer statistical significance. A p< 0.05 was considered statistically significant. Statistical analyses were per- formed by SPSS (version 22.0) software.

## RESULTS

There were 162 patients. Twenty-four patients were excluded due to absence of initial thorax CT. Among the remaining 138 patients, 5 did not want to participate in the study. Final 133 patients, (66 in group A and 67 in group B) who met the inclusion criteria were enrolled in the study (Figure 1).

Among the clinical features of patients, the male sex and age were statistically significantly different between the two groups (p= 0.004 and p< 0.001, respectively). Dyspnea was the most common symp- tom followed by fatigue. While there were no differ- ences between the groups in terms of comorbidities, the presence of smoking history was found to be significant between the groups. Fibrotic-like patterns were significantly more in patients with the male sex, older age, smoking history, high St. George’s Respiratory Questionnaire (SGRQ) total score, high dyspnea score, history of intensive care unit (ICU) admission, and reduced DLCO (Table 2).

A 4% decrease in oxygen saturation compared to the beginning of the test in the 6MWT was accepted as desaturation. Desaturation was detected in 48 (36.1%) of the patients. There was a significant differ- ence between the groups in terms of desaturation (p= 0.006).

Main thorax CT findings at admission were ground- glass opacities 125 (94%), consolidation 50 (37.6%), and intraparenchymal bands 11 (8.3%). There are no reticulations and traction bronchiectasis at admis- sion.

At the sixth month of COVID-19 pneumonia diagnosis, the most common radiographic findings were ground-glass opacities (46.6%), followed by normal CT (39%), reticulations (39%) and intraparenchymal bands (39%) (Table 3). Lung lesions of 29.3% of the patients were fully resolved at admission.

Figures 2 and 3 show thorax CT images of two cases with fibrotic-like pattern at follow-up.

Overall, DLCO % was predicted as 73.5% [IQR 61-88]. DLCO and 6MWD were significantly lower in the fibrotic-like pattern group (p< 0.001, p= 0.014, respectively). We found reduced DLCO after six months to be more common in patients with fibrotic- like pattern (Table 2).

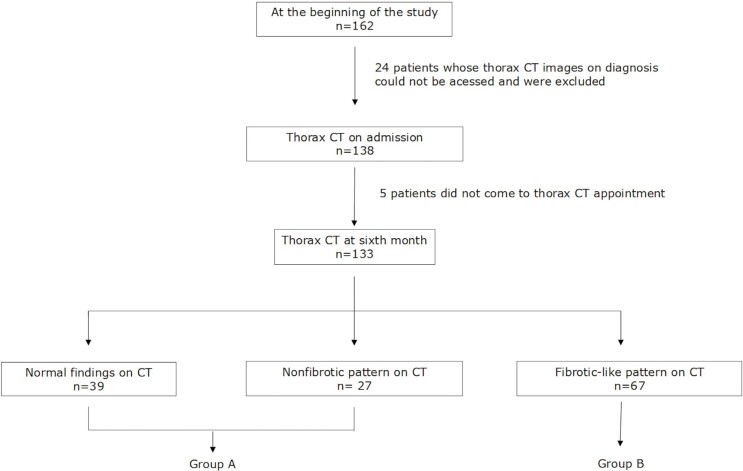

**Figure 1.** Flow chart of the study.

Presence of ground-glass opacities, reticulations and traction bronchiectasis correlated more strongly with reduction in diffusion capacity (r= -0.190 p= 0.043, r= -0.305 p= 0.001, r= -0.404 p< 0.001, respectively) (Table 4). Traction bronchiectasis did not correlate with FVC and walking distance. mMRC score ≥2 cor- related strongly with FVC, DLCO, and walking dis- tance (p= 0.003, 0.008, <0.001, respectively).

Multivariate binary logistic regression analysis between the fibrotic-like patterns and the risk factors in study group showed significant relationship between fibrotic-like changes and presence of smok- ing history and ICU admission, which suggests that smoking history and ICU admission are independent risk factors affecting fibrotic-like pattern (Table 5).

## DISCUSSION

The objective of this study was to determine the clinical features of patients during the sixth-month follow-up after a diagnosis with COVID-19, as well as to establish the risk variables associated with the development of a fibrotic-like pattern in these patients.

Advanced age is a significant risk factor for the devel- opment of pulmonary fibrosis following COVID-19

(9). Prior research had demonstrated that elderly individuals diagnosed with viral pneumonia are more susceptible to the development of pulmonary fibrosis (10,11). Farghaly et al. conducted case-con- trol research involving 64 patients, where they identi- fied a significant mortality rate among male patients and those aged 65 years or older who developed post-COVID fibrosis (12). In another study, male sex, age, baseline CT severity score, oxygen saturation, and duration of hospitalization were predictive fac- tors for fibrosis (13). In our study, mean age and rate of male patients were higher in group B compared to group A. With advanced age, cellular and humoral immune responses decrease, resulting in impaired control of viral replication and the development of an excessive inflammatory response, leading to poor outcomes in the course of the disease (14).

Smoking can result in the development of emphyse- ma, chronic bronchitis, and pulmonary fibrosis. Epidemiological study indicates that smokers are more likely to experience familial and sporadic idiopathic pulmonary fibrosis compared to non-smokers (15).

**Table:** 

**Table 2.** Demographic and clinical features between those with and without fibrotic-like patterns on thorax computed tomography				
	**Total n= 133**	**Group A n= 66**	**Group B n= 67**	**p**
Gender, n, (%)				
Male	75 (56.4)	29 (43.9)	46 (68.7)	**0.004**
Age ± SD	55.95 ± 12.42	51.94 ± 13.25	59.91 ± 10.19	**<0.001**
Smoking history, n, (%)				
Never smoker	83 (62.4)	49 (74.2)	34 (50.7)	**0.005**
Active and ex-smoker	50 (37.6)	17 (25.8)	33 (49.3)	**0.005**
Comorbidities, n, (%)				
Diabetes mellitus	37 (27.8)	19 (28.7)	18 (26.9)	0.191
Hypertension	40 (30.1)	15 (22.7)	25 (37.3)	0.166
COPD	8	2 (3)	6 (9)	0.350
Asthma	11 (8.3)	7 (10.6)	4	0.620
Heart disease	14 (10.5)	7 (10.6)	7 (10.4)	0.219
Place of COVID-19 pneumonia treatment, n, (%)				
ICU admission	34 (25.6)	9 (13.6)	25 (37.3)	**0.020**
Hospital wards	54 (40.6)	28 (42.4)	26 (38.8)	0.671
Outpatient	46 (34.6)	29 (43.9)	16 (23.9)	**0.015**
SGRQ total score, median [IQR]				
On diagnosis	30 [17-50.8]	22.52 [13.14-39.32]	33.62 [22.69-52.57]	**<0.001**
At sixth month	18.76 [8.47-31.42]	16.11 [5.09-28.25]	23.90 [16.34-33.69]	**0.002**
Laboratory on diagnosis, median [IQR]				
Lymphocyte	1150 [945-1550]	2215 [1682.5-2672.5]	2270 [1800-2920]	0.099
D-dimer	320 [176.5-534.5]	251.5 [152.5-445.2]	378 [188-585]	0.062
Fibrinogen	4.5 [3.58-5.52]	4.47 [3.69-5.44]	4.5 [3.4-5.6]	0.734
Ferritin	194 [74.9-450]	159.75 [67-440.2]	231 [98-450]	0.297
CRP	24 [7.3-66]	19.58 [6.45-54.4]	34 [9.5-88.8]	0.052
Symptoms at sixth month, n, (%)				
Asymptomatic	29 (21.8)	19 (28.8)	10 (14.9)	0.053
Dyspnea	72 (54.1)	30 (45.5)	42 (62.7)	**0.046**
Cough	22 (16.5)	7 (10.6)	15 (22.4)	0.067
Fatique	55 (41.4)	28 (42.4)	27 (40.3)	0.803
PFTs at sixth month				
FVC, % predicted, median [IQR]	94 [82-111]	103 [85.5-115]	89.5 [80.75-99.5]	**0.004**
	n= 115	n= 53	n= 62	
DLCO, % predicted, median [IQR]	73.5 [61-88]	81 [69-93.75]	68[55.5-79.2]	**<0.001**
	n= 114	n= 52	n= 62	
TLC, % predicted, median [IQR]	101 [88.2-120]	108 [96-125]	95 [85-114]	**0.004**
	n= 100	n= 49	n= 51	
6MWD, meter, median [IQR]	540 [465-620]	555 [492.5-650]	500 [440-600]	**0.014**
	n= 131	n= 64	n= 67	
6MWT, desaturation, n, (%)	48 (36.1)	16 (25)	32 (47.8)	**0.006**
	n= 131	n= 64	n= 67	
mMRC score ≥2, n, (%)	43 (32.4)	14 (21.2)	29 (43.3)	**0.029**
COPD: Chronic obstructive pulmonary disease, ICU: Intensive care unit, SGRQ: Saint George respiratory questionnare, CRP: C-reactive protein, PFTs: Pulmonary function tests, FVC: Forced vital capacity, DLCO: Lung diffusing capacity for cabon-monoxide, TLC: Total lung capacity, 6MWD: Six minute walking distance, 6MWT: Six minute walking test, mMRC: Modified medical research council.				

**Table:** 

**Table 3.** Prevalence of thorax computed tomography abnormalities at sixth month after COVID-19 diagnosis				
	**Total n= 133**		**non-Fibrotic pattern n= 27**	**Fibrotic-like pattern n= 67**
No abnormality	39	(29.3)		
non-Fibrotic abnormalities				
Intraparenchymal bands	45	(33.8)	6 (22.2)	39 (58.2)
Ground-glass opacities	62	(46.6)	27 (100)	35 (52.2)
Consolidation	5	(3.8)	2 (7.4)	3 (4.5)
Fibrotic abnormalities				
Reticulations	39	(29.3)	0	39 (58.2)
Honey-combing		0	0	0
Traction bronchiectasis	28	(21.1)	0	28 (41.8)
Results were given as n (%).

**Figure 2.** A 68-year-old male patient **(A)** bilateral ground-glass opacities on thorax CT at admission **(B)** at six months after discharge, most lesions had resolved, but subpleural reticulations persisted.

**Figure 3.** A 73-year-old male patient was treated in the ICU **(A)** bilateral ground-glass opacities and consolidations on thorax CT at admission **(B)** at six months after discharge, subpleural reticulations, traction bronchiectasis persisted.

**Table:** 

**Table 4.** Pearson point biserial correlation coefficients of CT pattern and mMRC ≥2 with FVC, DLCO, walking distance on 6MWT								
		**FVC**			**DLCO**		**6MWD, m**	
CT pattern	**r**	**p**		**r**	**p**		**r**	**p**
Ground glass opacities	-0.114	0.227		-0.190	**0.043**		-0.188	0.031
Reticulation	-0.280	**0.002**		-0.305	**0.001**		-0.175	**0.046**
Traction bronchiectasis	-0.130	0.165		-0.404	**<0.001**		-0.081	0.335
mMRC ≥2	-0.274	**0.003**		-0.248	**0.008**		-0.430	**<0.001**
FVC: Forced vital capacity, DLCO: Lung diffusing capacity for cabon-monoxide, 6MWD: Six minute walking distance, m: Meter, CT: Computed tomography, r: Correlation co-efficients, mMRC: Modified medical research council.								

**Table:** 

**Table 5.** Association between independent variables of interest in fibrotic-like patterns in multivariable logistic regression			
**Variable**	**OR**	**95% Cl**	**p**
Age ≥65	1.480	0.554-3.955	0.434
Gender, Male	2.262	0.923-5.540	0.074
Smoking	2.740	1.134-6.620	**0.025**
ICU admission	4.698	1.463-15.083	**0.009**
Inpatient clinic admission	1.956	0.767-4.986	0.160
Presence of a comorbidity	0.619	0.257-1.490	0.285
Lymphopenia	2.557	0.992-6.591	0.052
CRP	0.713	0.221-2.297	0.570
D-dimer	1.875	0.781-4.503	0.159
Ferritin	0.521	0.186-1.459	0.215
Fibrinogen	0.893	0.348-2.289	0.814
OR: Odds ratio, CI: Confidence interval, ICU: Intensive care unit, CRP: C-reactive protein.

Smoking causes long-term oxidative stress, inflamma- tory cytokines, and interstitial lung fibrosis (9). A com- prehensive investigation has revealed that smokers had a 1.4-fold higher probability of suffering severe symptoms of COVID-19. In addition, smokers had a

2.4 times higher likelihood of needing admission to an ICU, requiring mechanical ventilation, or facing a higher risk of mortality, compared to non-smokers

(16). Another study revealed that current smoking has the greatest influence on disease progression among the variables studied in COVID-19 (17). A study evalu- ating the smoking status of 1099 patients observed that a higher proportion of smokers were discovered among patients with severe symptoms and those who needed ICU follow-up. However, the study did not analyze the relationship between the severity of COVID-19 and smoking status (18). In our study, simi- lar to the literature, it was found that fibrotic-like changes were more prevalent in patients with a smok- ing history compared to non-smokers.

Farghaly et al. reported that the patients most suscep- tible to developing fibrosis were those who were

admitted to the ICU and needed mechanical ventila- tor support as a result of COVID-19. Similarly, our investigation revealed that history of ICU admission was determined to be an independent risk factor for the development of a fibrotic-like pattern (12). In a study evaluating the two-year follow-up of 348 COVID-19 patients, subjects who had been admitted to the ICU had a higher risk of developing a fibrotic- like pattern (19). Similarly, we found that ICU admis- sions were more frequent in the group developing a fibrotic-like pattern, and ICU admission was identi- fied as an independent risk factor for the develop- ment of a fibrotic-like pattern.

Previous waves of the COVID-19 pandemic have repeatedly demonstrated a significant occurrence of pulmonary diffusion impairments, with reported rates varying between 11% and 34% (2,20,21). Severe cases of COVID-19 have a more severe decrease in diffusion capacity early after the acute phase of the infection, in comparison to moderate cases. Acute respiratory distress syndrome, which is the most prevalent consequence of COVID-19, frequently

leads to this impairment. Research has indicated that the decrease in diffusion capacity is persistent, and this is partly due to irreversible damage to the endothelial cells (22,23). A meta-analysis revealed that during previous epidemics of coronaviruses such as Middle East respiratory syndrome and severe acute respiratory syndrome (SARS), approximately 10-34% of the patients had a decrease in their diffusion capac- ity when assessed one year later (21). In the same meta-analysis, comparing patients with and without ICU admission, FVC and DLCO values were found to be relatively lower in the group with ICU admission

(21). It should be noted that in patients admitted to the ICU, decreased respiratory function parameters may be detected due to the developing muscle weakness. Recent investigations have shown that COVID-19 primarily impacts the lungs, causing various lung conditions such as diffuse destruction of the alveolar epithelium, injury or bleeding in the capillaries, crea- tion of hyaline membranes, increased fibrous tissue in the alveolar septa, and consolidation of the lungs. In the study conducted by Park et al., they found that 15.5-43.6% of patients who were observed for about two years due to SARS had a decrease in DLCO, which was the most common functional abnormality

(24). In a study conducted by Liu et al., it was observed that lung consolidations in COVID-19 patients recovered more rapidly compared to other types of lesions, around one month following dis- charge (25). In a study conducted approximately three years later evaluating COVID-19 patients, it was observed that although there was a gradual improve- ment, the group that developed fibrosis had lower DLCO, shorter walking distances, and more frequent respiratory symptoms compared to the control group

(26). Compatible with literature findings, the group with a fibrotic-like pattern in the present study exhib- ited lower DLCO values, shorter walking distances, and more frequent respiratory symptoms.

Although SGRQ scores at the six-month follow-up were better in both groups compared to the disease period, patients in the fibrotic-like pattern group had worse scores than those in the non-fibrotic group. The most common persistent symptom was dyspnea, and according to the MRC dyspnea scale, MRC scores were higher in the fibrotic-like pattern group. This situation can be explained by the lower diffusing capacity observed in this group.

There are limitations in our study. First, the absence of baseline pulmonary function test results prior to

the illness poses a challenge in comparing pre-illness and post-illness outcomes. Additionally, this cross- sectional analysis offers only a brief follow-up peri- od, leaving the long-term trajectory of lung function after hospital discharge unexplored and in need of further study. The follow-up duration was relatively short, and extended CT follow-up is necessary to assess whether the observed reticular patterns pro- gress to irreversible fibrosis.

## CONCLUSION

In conclusion, our study highlights that among the discharged COVID-19 survivors, impaired diffusion capacity is the most frequent lung function abnormality, followed by restrictive ventilatory defects, both of which correlate with disease severity. Routine clinical follow-up for certain recovered patients, particularly those with severe cases, should include comprehensive pulmonary function tests, encompassing not only spirometry but also diffusion capacity assessment.

Six months after discharge, the most common imag- ing findings in COVID-19 survivors were ground- glass opacities, intraparenchymal bands, and reticu- lations. Smoking history and ICU admission emerged as key risk factors for incomplete radiological resolu- tion. Fibrotic-like abnormalities were notably preva- lent at six months and were linked to impaired lung diffusion. Further research is essential to understand the long-term progression of these fibrotic-like changes and their implications.

**Ethical Committee Approval:** This study was approved by the Ankara University Faculty of Medicine Human Research Ethics Committee (Decision no: I1-15-21, Date: 14.01.2021).

### CONFLICT of INTEREST

The authors declare that they have no conflict of interest.

## AUTHORSHIP CONTRIBUTIONS

Concept/Design: MÖK, AGK, AK Analys/Interpretation: MÖK, AGK Data acqusition: ÖVY, SND, MÖK

Writing: MÖK, AGK, FA, SE, AÇ, ÇU, NDA, İA, GÇ, KOM, OK, ÖÖK, ÖY, SS, AK

Clinical Revision: AGK, FA, SE, AÇ, ÇU, NDA, İA, GÇ, KOM, OK, ÖÖK, ÖY, SS, AK

Final Approval: All of authors

## REFERENCES

1. *
  Öz M, Erol S, Gürün Kaya A, Işık Ö, Çiftci F, Çınar G, et al. Recovery from respiratory failure in patients with corona- virus disease 2019. Thorac Res Pract 2023; 25(1): 26-34. https://doi.org/10.5152/ThoracResPract.2023.23001*
2. *
  van Raaij BFM, Stöger JL, Hinnen C, Penfornis KM, de Jong CMM, Klok FA, et al. Fibrotic-like abnormalities notably prevalent one year after hospitalization with COVID-19. Respir Med Res 2022; 82: 100973. https://doi.org/10.1016/j.resmer.2022.100973*
3. *
  Li X, Shen C, Wang L, Majumder S, Zhang D, Deen MJ, et al. Pulmonary fibrosis and its related factors in discharged patients with new corona virus pneumonia: A cohort study. Respir Res 2021; 22(1): 203. https://doi.org/10.1186/s12931-021-01798-6*
4. *
  Huang Y, Tan C, Wu J, Chen M, Wang Z, Luo L, et al. Impact of coronavirus disease 2019 on pulmonary func- tion in early convalescence phase. Respir Res 2020; 21(1): 163. https://doi.org/10.1186/s12931-020-01429-6*
5. *
  McGroder CF, Zhang D, Choudhury MA, Salvatore MM, D’Souza BM, Hoffman EA, et al. Pulmonary fibrosis 4 months after COVID-19 is associated with severity of ill- ness and blood leucocyte telomere length. Thorax 2021; 76(12): 1242-5. https://doi.org/10.1136/thorax-jnl-2021-217031*
6. *
  Miller MR, Hankinson J, Brusasco V, Burgos F, Casaburi R, Coates A, et al. Standardisation of spirometry. Eur Respir J 2005; 26(2): 319-38. https://doi.org/10.1183/09031936.05.00034805*
7. *
  Celli BR, MacNee W. Standards for the diagnosis and treatment of patients with COPD: A summary of the ATS/ ERS position paper. Eur Respir J 2004; 23(6): 932-46. https://doi.org/10.1183/09031936.04.00014304*
8. *
  ATS Committee on Proficiency Standards for Clinical Pulmonary Function Laboratories. ATS statement: Guidelines for the six-minute walk test. Am J Respir Crit Care Med 2002; 166(1): 111-7. https://doi.org/10.1164/ajrccm.166.1.at1102*
9. *
  Ojo AS, Balogun SA, Williams OT, Ojo OS. Pulmonary fibrosis in COVID-19 survivors: Predictive factors and risk reduction strategies. Pulm Med 2020; 2020: 6175964. https://doi.org/10.1155/2020/6175964*
10. *
  Sansone A, Mollaioli D, Ciocca G, Limoncin E, Colonnello E, Vena W, et al. Addressing male sexual and reproductive health in the wake of COVID-19 outbreak. J Endocrinol Invest 2021; 44(2): 223-31. https://doi.org/10.1007/s40618-020-01350-1*
11. *
  Wong KT, Antonio GE, Hui DS, Ho C, Chan PN, Ng WH, et al. Severe acute respiratory syndrome: Thin-section computed tomography features, temporal changes, and clinicoradiologic correlation during the convalescent peri- od. J Comput Assist Tomogr 2004; 28(6): 790-5. https://doi.org/10.1097/00004728-200411000-00010*
12. *
  Farghaly S, Badedi M, Ibrahim R, Sadhan MH, Alamoudi A, Alnami A, et al. Clinical characteristics and outcomes of post-COVID-19 pulmonary fibrosis: A case-control study. Medicine (Baltimore) 2022; 101(3): e28639. https://doi.org/10.1097/MD.0000000000028639*
13. *
  Sarıoğlu N, Aksu GD, Çoban H, Bülbül E, Demirpolat G, Arslan AT, et al. Clinical and radiological outcomes of long COVID: Is the post-COVID fibrosis common? Tuberk Toraks 2023; 71(1): 48-57. https://doi.org/10.5578/tt.20239907*
14. *
  Zhou F, Yu T, Du R, Fan G, Liu Y, Liu Z, et al. Clinical course and risk factors for mortality of adult inpatients with COVID-19 in Wuhan, China: A retrospective cohort study. Lancet 2020; 395(10229): 1054-62. https://doi.org/10.1016/S0140-6736(20)30566-3*
15. *
  Taskar VS, Coultas DB. Is idiopathic pulmonary fibrosis an environmental disease? Proc Am Thorac Soc 2006; 3(4): 293-8. https://doi.org/10.1513/pats.200512-131TK*
16. *
  Vardavas CI, Nikitara K. COVID-19 and smoking: A sys- tematic review of the evidence. Tob Induc Dis 2020; 18:20. https://doi.org/10.18332/tid/119324*
17. *
  Liu W, Tao ZW, Wang L, Yuan ML, Liu K, Zhou L, et al. Analysis of factors associated with disease outcomes in hospitalized patients with 2019 novel coronavirus disease. Chin Med J (Engl) 2020; 133(9): 1032-8. https://doi.org/10.1097/CM9.0000000000000775*
18. *
  Guan WJ, Ni ZY, Hu Y, Liang WH, Ou CQ, He JX, et al. Clinical characteristics of coronavirus disease 2019 in China. N Engl J Med 2020; 382(18): 1708-20. https://doi.org/10.1056/NEJMoa2002032*
19. *
  Ribeiro Carvalho CR, Lamas CA, Visani de Luna LA, Chate RC, Salge JM, Yamada Sawamura MV, et al. Post-COVID-19 respiratory sequelae two years after hospitalization: An ambidirectional study. Lancet Reg Health Am 2024; 33: 100733. https://doi.org/10.1016/j.lana.2024.100733*
20. *
  Hui DS, Joynt GM, Wong KT, Gomersall CD, Li TS, Antonio G, et al. Impact of severe acute respiratory syndrome (SARS) on pulmonary function, functional capacity and quality of life in a cohort of survivors. Thorax 2005; 60(5): 401-9. https://doi.org/10.1136/thx.2004.030205*
21. *
  Ahmed H, Patel K, Greenwood DC, Halpin S, Lewthwaite P, Salawu A, et al. Long-term clinical outcomes in survivors of severe acute respiratory syndrome and Middle East respiratory syndrome coronavirus outbreaks after hospi- talisation or ICU admission: A systematic review and meta-analysis. J Rehabil Med 2020; 52(5): jrm00063. https://doi.org/10.2340/16501977-2694*
22. *
  Huang L, Yao Q, Gu X, Wang Q, Ren L, Wang Y, et al. 1-year outcomes in hospital survivors with COVID-19: A longitudinal cohort study. Lancet 2021; 398(10302): 747-58. https://doi.org/10.1016/S0140-6736(21)01755-4*
23. *
  Wu X, Liu X, Zhou Y, Yu H, Li R, Zhan Q, et al. 3-month, 6-month, 9-month, and 12-month respiratory outcomes in patients following COVID-19-related hospitalisation: A prospective study. Lancet Respir Med 2021; 9(7): 747-54. https://doi.org/10.1016/S2213-2600(21)00174-0*
24. *
  Park WB, Jun KI, Kim G, Choi JP, Rhee JY, Cheon S, et al. Correlation between pneumonia severity and pulmonary complications in Middle East respiratory syndrome. J Korean Med Sci 2018; 33(24): e169. https://doi.org/10.3346/jkms.2018.33.e169*
25. *
  Liu C, Ye L, Xia R, Zheng X, Yuan C, Wang Z, et al. Chest computed tomography and clinical follow-up of dis- charged patients with COVID-19 in Wenzhou City, Zhejiang, China. Ann Am Thorac Soc 2020; 17(10): 1231-7. https://doi.org/10.1513/AnnalsATS.202004-324OC*
26. *
  Han X, Chen L, Guo L, Wu L, Alwalid O, Liu J, et al. Long- term radiological and pulmonary function abnormalities at 3 years after COVID-19 hospitalisation: A longitudinal cohort study. Eur Respir J 2024; 64(1): 2301612. https://doi.org/10.1183/13993003.01612-2023*

